# Pituitary involvement in ANCA-associated vasculitis: Case report and literature review

**DOI:** 10.1097/MD.0000000000047229

**Published:** 2026-01-23

**Authors:** Xin Chen, YiFan Xie, MengLi Xu, Chao Wang, Qi Cheng, Jing Xue, Yan Du

**Affiliations:** a Department of Rheumatology, Affiliated Jinhua Hospital, Zhejiang University School of Medicine, Jinhua, China; b Department of Rheumatology, The Second Affiliated Hospital of Zhejiang University School of Medicine, Hangzhou, China; c Department of Nephrology, The Third Affiliated Hospital of Zhejiang Chinese Medical University, Hangzhou, China; d Department of Radiology, The Second Affiliated Hospital, Zhejiang University School of Medicine, Hangzhou, China.

**Keywords:** ANCA-associated vasculitis, hypophysitis, pituitary

## Abstract

The antineutrophil cytoplasmic antibody (ANCA)-associated vasculitis (AAV) is an rare autoimmune condition characterized by inflammatory cell infiltration causing necrosis of small blood vessels. Pituitary involvement is uncommon in AAV and it can occur at any point during AAV. The main clinical manifestations are central diabetes insipidus and panhypopituitarism. In this article, we describe 2 cases of pituitary involvement of AAV, pituitary dysfunction presented as initial symptom in 1 patient and developed over the course of the diseases in the other patient. Both of them had positive ANCA titers and myeloperoxidase positive antibodies. Two patients both had an enlarged pituitary, shown by magnetic resonance images, 1 patient also had Rathke’s cleft cyst. After treatment with intravenous steroids, immunosuppressant, and biological agent, clinical symptoms as well as pituitary imaging were alleviated significantly. We go over a brief review of the currently available literature on pituitary involvement in AAV. The literature review identified 42.55% exhibited pituitary dysfunction at disease onset, commonly presenting with diabetes insipidus and deficiencies in pituitary hormones such as follicle-stimulating hormone-luteinizing hormone, thyrotropin, adrenocorticotropic hormone, and growth hormone. Beyond pituitary involvement, other organ lesions most frequently affect the ear, nose, throat, eye, pulmonary, and kidney systems. Pituitary magnetic resonance imaging was pivotal in lesion identification, frequently showing pituitary enlargement, sellar masses, and optic chiasm compression. The majority of patients achieved systemic disease remission or improvement during follow-up, although 52.17% continued to exhibit persistent pituitary dysfunction. The latest EULAR and KDIGO guidelines recommend glucocorticoids combined with immunosuppressive agents such as rituximab or cyclophosphamide for initial AAV treatment. Our cases demonstrated the effectiveness of early aggressive therapy with glucocorticoids combined with mycophenolate mofetil or rituximab in achieving disease remission and improving pituitary function.

## 1. Introduction

Following the initial discovery of the anti-neutrophil cytoplasmic antibodies (ANCAs) in 1982^[1]^, ANCA-associated vasculitis (AAV) was established as a disease entity distinct from other vasculitis. AAV is now recognized as a systemic vasculitis which affects small vessels and is accompanied by the presence of ANCAs in the serum. The major antigens targeted by these ANCAs are myeloperoxidase (MPO) and proteinase 3 (PR3). AAV refers most often to 3 distinct but overlapping diseases: microscopic polyangiitis (MPA), granulomatosis with polyangiitis (GPA) and eosinophilic granulomatosis with polyangiitis.^[2]^ The clinical characters of AAV include a skin rash as well as fulminant multisystem disease. Typical features of MPA include severe renal and pulmonary disease. Patients with GPA have a predilection for ear, nose, and throat, upper respiratory tract, lower respiratory tract, and kidney disease. Patients with eosinophilic granulomatosis with polyangiitis typically have a back-ground of peripheral blood eosinophilia, asthma, and nasal polyposis.^[3]^

Central nervous system involvement was reported in 7% to 11% of patients with GPA and 9.8% in MPA.^[4]^ The pituitary gland, the meninges, and the cerebral vasculature are the major structures involved in AAV. However, pituitary dysfunction (PD) is a rare clinical symptom in AAV. Several retrospective study reported that PD occurred in 0.8% to 3.9% of GPA.^[5–8]^ And Li et al^[4]^ reported that 2% AAV patients were found to have pituitary involvement. There are few reports of pituitary dysfunction as the primary manifestation in patients with AAV. Here we present 2 rare cases of AAV who primarily presented with pituitary dysfunction and compare them with a systematic review of all published cases with the same disease.

## 2. Case description

### 2.1. Case 1

**Figure 1.**
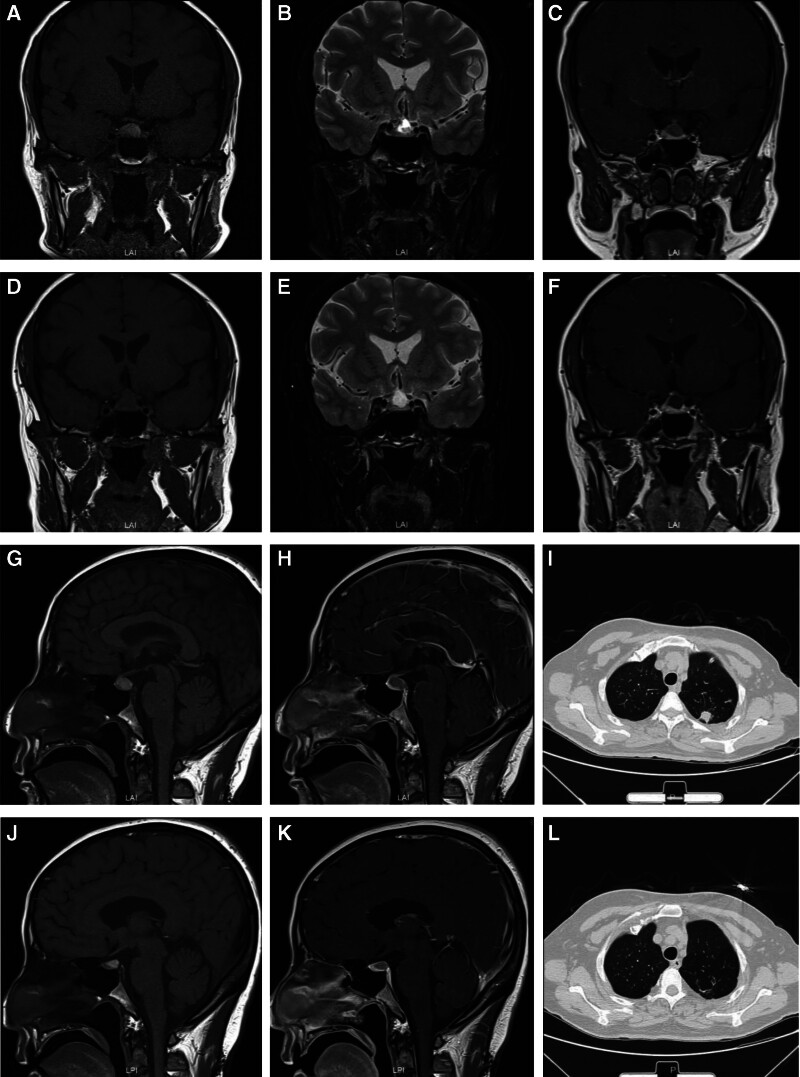
Imaging findings in case 1. Pituitary MRI T1-weighted (A, G): shows high signal intensity in the sellar region, suggestive of pituitary enlargement (12 mm × 15 mm), with Rathke’s cleft cyst (10 mm × 11 mm), no obvious compression of the optic chiasm; T2-weighted (B): shows high signal intensity of Rathke’s cleft cyst; enhanced pituitary MRI (C, H): no significant enhancement observed in the pituitary gland; chest CT scan (I): reveals a subpleural nodule in the left upper lung, possibly representing a chronic inflammatory nodule; follow-up pituitary MRI (D–F, J, K): the pituitary size (11 mm × 15 mm) appears the same compared to previous scans, Rathke’s cleft cyst appears the same size (9 mm × 12 mm) as compared to previous scans; follow-up chest CT scan (L): demonstrates resolution of the subpleural nodule. MRI = magnetic resonance imaging, CT = computerized tomography.

A 52-year-old female who had headache, dizzy, fever (T_max_ 38.2), polyuria–polydipsia syndrome with nycturia, and a daily water intake up to 4 L was admitted in the department of Endocrine. Laboratory investigation showed white blood count 8.5 × 10^9^/L, hemoglobin 111 g/L, platelet count 317 × 10^9^/L. Urine routine, liver function and renal function tests were normal. Erythrocyte sedimentation rate (ESR) amounted to 83 mm/h (normal range, 0–20 mm/h). C reactive protein amounted to 63.8 mg/L (normal range, <10 mg/L). Anti tuberculosis antibody, anti nuclear antibody and anti ENA peptides antibody were negative. Antineutrophil cytoplasmic antibody in a cytoplastic pattern (c-ANCA) was found with positive PR3 subtype. Serum levels of thyroid hormone, sex hormone and growing hormone (GH) were normal. A pituitary MRI demonstrated pituitary enlargement (12 mm × 15 mm) with Rathke’s cleft cyst (10 mm × 11 mm; Fig. 1A–C, G, H). Chest CT implied lung infection and an subpleural inflammatory nodule (Fig. 1I). Bronchoalveolar lavage fluid (BALF) test showed Klebsiella pneumoniae. The initial therapy was cephalosporin as well as desmopressin. One week later, the symptoms did not abate, and she had pain in the bilateral calf muscles and tinnitus. After consultation with a rheumatologist, the patient was considered to have fever, elevated serum inflammatory markers, normal eosinophil count, positive c-ANCA and PR3 antibodies, and chest imaging suggestive of pulmonary inflammatory nodules. These findings meet the 2022 ACR/EULAR GPA classification criteria, leading to a diagnosis of GPA. MP at 40 mg/d and mycophenolate mofetil (MMF) at 1.0 g/d were given to the patient, the fever, pain, and polyuria–polydipsia resolved quickly. However, Rathke’s cleft cyst did not shrink (9 mm × 12 mm), and there was no significant change in the pituitary size (11 mm × 15 mm; Fig. 1D–F, J, K). Chest CT demonstrates resolution of the subpleural nodule (Fig. 1L). One year after her diagnosis as GPA, she received pituitary surgery because of Rathke’s cleft cyst. After 3 years of follow-up, GPA remained in remission with low doses of prednisone (2.5 mg/d) as maintenance treatment.

### 2.2. Case 2

**Figure 2.**
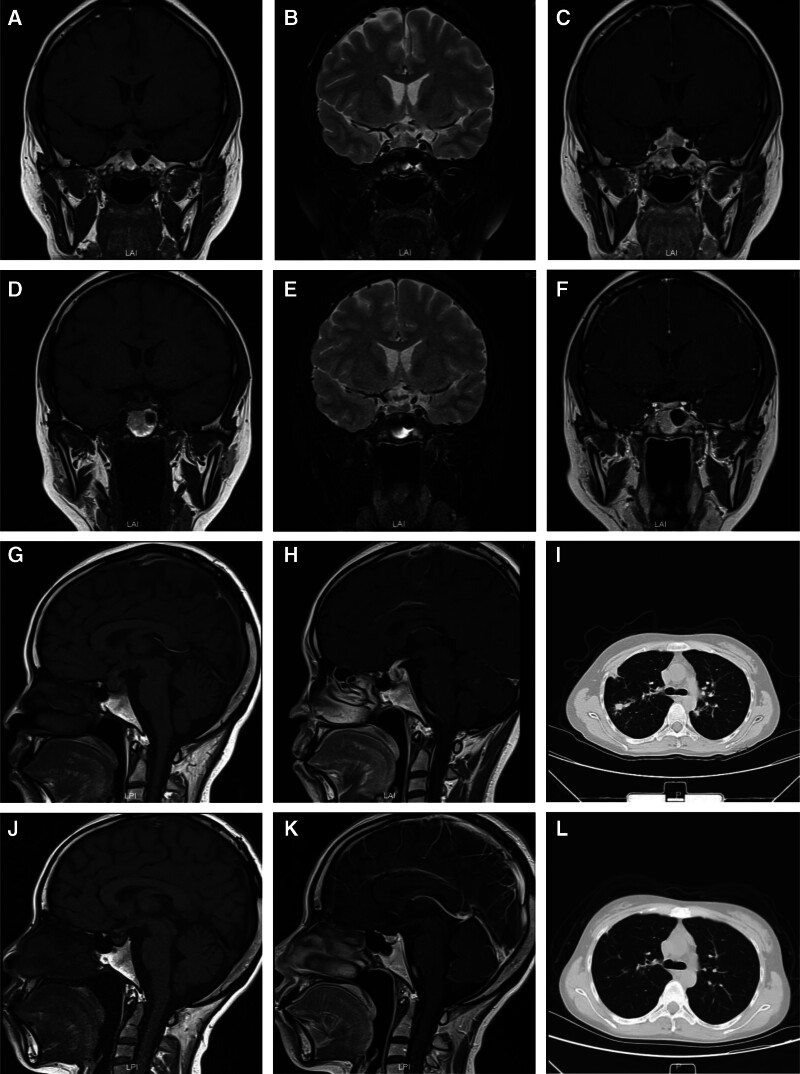
Imaging findings in case 2. Pituitary MRI T1-weighted (A, G): enlargement and thickening of the pituitary gland (11 mm × 10 mm) and infundibulum with abnormal signal intensity; T2-weighted (B): shows moderate signal intensity of the pituitary gland; enhanced pituitary MRI (C, H): heterogeneous contrast enhancement within the pituitary; chest CT scan (I): reveals multiple mass-like shadows in the lungs; follow-up pituitary MRI (D–F, J, K): showed a smaller pituitary gland (10 mm × 6 mm) compared to previous scans; follow-up chest CT scan (L): demonstrates resolution of the masses. MRI = magnetic resonance imaging, CT = computerized tomography.

A 26-year old female presented with tiredness and weight loss. Local hospital laboratory investigation showed white blood count 8.7 × 10^9^/L with 79% neutrophil, hemoglobin 117 g/L, platelet count 365 × 10^9^/L. Urine routine showed red blood cells 3842/L, protein 2+, renal function tests showed creatinine 165 μmol/L (normal range 41–72 μmol/L), ESR 112 mm/h, CRP 18 mg/L, ANA and ENA antibodies were negative, p-ANCA and anti-MPO antibodies were positive. The patient presented with nonspecific symptoms, elevated serum inflammatory markers, normal eosinophil count, positive p-ANCA and MPO antibodies, meeting the 2022 ACR/EULAR MPA classification criteria, leading to a diagnosis of MPA. Induction to remission treatment with high-dose methylprednisolone, (MP, 320 mg/d for 3 days and then 40 mg/d for 6 weeks) followed by a tapering regimen and MMF 1 g/d were prescribed. Three years later after the diagnosis of MPA, the patient started with headache, cough, left eye pain, fever (T_max_ 39.1), polyuria–polydipsia syndrome with nycturia, and a daily water in take up to 6 L. An enlarged pituitary gland (11 mm × 10 mm) with heterogeneous contrast enhancement on MRI studies was observed (Fig. 2A–C, G, H) and multiple mass shadows were found on chest computerized tomography (CT; Fig. 2I). Serum sex hormone showed prolactin (PRL) 25.4 ng/mL. Serum levels of luteinizing hormone, follicle-stimulating hormone, GH and thyroid-stimulating hormone were normal. Dehydration test with vasopression measurement confirmed diabetes insipidus (DI). Considering the MRI findings and central DI, the patient was diagnosed with infundibulum-neurohypophysitis and treated with desmopressin. At this point, MPA was in recurrence and the ESR and CRP were elevated (47 mm/h and 74.5 mg/L). MP was used at 80 mg/d for 1 week and then MP was reduced back to 40 mg/d, with administraiton of Rituximab. The symptoms including headaches, cough and polyuria–polydipsia syndrome with nycturia were all relieved. Blood tests showed normal ESR and CRP, and negative ANCA. After 3 years of follow-up, pituitary MRI revealed a smaller pituitary than before (10 mm × 6 mm; Fig. 2D–F, J, K). Follow-up chest CT scan demonstrates resolution of the masses (Fig. 2L). MPA remained in remission receiving low doses of prednisone (7.5 mg/d) as maintenance treatment.

Table 1 summarizes the clinical and laboratory findings, treatment details, and outcomes for the 2 cases of pituitary dysfunction due to AAV in this study.

**Table 1:** Clinical and laboratory findings, treatment, and outcomes of patients with pituitary dysfunction due to AAV in this study.

Articles	Age (yr)	Sex	Time of pituitary problems	Pituitary dysfunction	Other organs involvement	ANCA patterns and titers	Treatment	Final outcome
1	51	F	At onset of AAV	Enlargement of pituitary with Rathke’s cleft cyst, protruding to the sellar	Lung, muscle, eyes	PR3-ANCA 90.68 (Normal < 20 U/mL)	MP + MMF	Resolved
2	26	F	3 yr	Enlargement of pituitary with abnormal enhancement, irregular margin, thickening of pituitary stalk	Lung, kidney	MPO-ANCA 40.15 (Normal < 20 U/mL)	MP + MMF, replaced with MP + Riuximab at the point of PD	Resolved

AAV = ANCA-associated vasculitis, ACL = anti-cardiolipin, anti-CCP = anti-cyclic citrullinated peptid, Anti-dsDNA = anti-double strand DNA, APLAs = antiphospholipid antibodies, ASA = aspirin, AZA = azathioprine, BE = both eyes, CYC = cyclophosphamide, HCQ = hydroxychloroquine, IVIG = intravenous immunoglobulin, IVMP = intravenous methylprednisolone, LAC = lupus anticoagulant, LE = left eye, MMF = mycophenolate mofetil, MTX = methotrexate, RE = right eye, ß2-GPI = anti-β2 glycoprotein 1.

## 3. Literature review and discussion

We conducted a systematic search of the National Library of Medicine PubMed database, Web of Science, and Scopus using the key terms “(ANCA-associated vasculitis) OR (microscopic polyangiitis) OR (granulomatosis with polyangiitis) OR (eosinophilic granulomatosis with polyangiitis)” AND “(Pituitary Dysfunction) OR (Hypophysitis) OR (infundibulo-hypophysitis)”. Based on this search, we reviewed all English-language reported cases. Among 50 articles published between 1978 and 2024, we identified a total of 94 patients with AAV. Notably, 40 out of these 94 patients (42.55%)^[4-31]^ exhibited pituitary dysfunction at the initial diagnosis of AAV, which aligns with the frequency observed in our 2 cases.

The clinical characteristics, hormonal profiles, ANCA patterns, treatment regimens, and outcomes for these patients are summarized in Table 2.

**Table 2:** The clinical characteristics, hormonal profiles, ANCA patterns, treatment regimens, and outcomes for AAV combine with PD.

Clinical feature	Statistic		Clinical feature	Statistic	
Demographics			Time of PD Incidence in AAV patients		
Age of PD (medium [min–max])	45 (13–80)		Time of pituitary problems after AAV diagnosed (medium [min–max])	1 (0–180)	
Sex (F/M)	68/26		PD concomitant with AAV	40 (42.55%)	

Other organs involvement	n	%	Treatment	n	%
ENT	62	65.96%	GC	92	97.87%
Eye	35	37.23%	PDGC	20	21.28%
Peripheral neuropathy	11	11.70%	IVIG	6	6.38%
CNS	15	15.96%	Plasma exchange	2	2.13%
HCP	8	8.51%	CTX	76	80.85%
CVST	2	2.13%	MTX	15	15.96%
Pulmonary	32	34.04%	LEF	1	1.06%
Kidney	22	23.40%	Aza	11	11.70%
Cardiac	1	1.06%	MMF	11	11.70%
Skin	18	19.15%	FK/CsA	5	5.32%
Joints	12	12.77%	TNFi	9	9.57%
Myalgias	5	5.32%	RTX	18	19.15%

Pituitary dysfunction	n/all	%	Systemic diseases response	n	%
DI	80/90	88.89%	Remission	57	64.04%
FSH-LH	37/75	49.33%	Improved	27	30.34%
TSH	34/77	44.16%	Relapse	2	2.25%
ACTH	33/63	52.38%	Death	3	3.37%
GH	12/59	20.34%			
PRL	33/77	42.86%			

ANCA patterns	n/all	%	Pituitary function post treatment	n	%
cANCA/PR3	44/67	65.67%	Recovered	13	18.06%
pANCA/MPO	14/67	20.90%	Partial recovered	19	26.39%
Negative	9/67	13.43%	Persistent	37	51.39%
			Death	3	4.17%

AAV = ANCA-associated vasculitis, ACTH = adrenocorticotropic hormone, Aza = azathioprine, CNS = central nervous system, CTX = cyclophosphamide, CVST = cerebral venous sinus thrombosis, DI = diabetes insipidus, ENT = ear, nose, throat, FK/CsA = tacrolimus, cyclosporin A, FSH-LH = follicle-stimulating hormone-luteinizing hormone, GC = glucocorticoids, GH = growth hormone, HCP = hypertrophic cranial pachymeningitis, IVIG = intravenous immunoglobulin, LEF = leflunomide, MMF = mycophenolate mofetil, MTX = methotrexate, PDGC = pulse-dose glucocorticoids, PRL = prolactin, RTX = rituximab, TNFi = tumor necrosis factor inhibitor, TSH = thyroid-stimulating hormone.

Regarding the pathology of AAV with PD, small vessel vasculitis is considered the primary contributor to the observed clinical lesions. However, the presence of pituitary dysfunction at disease onset is uncommon; only 15 patients underwent pituitary biopsy during their initial diagnosis.^[4,6,8,9,14,20,26,30,32-38]^ The main histopathological findings included granulomatous inflammation,^[4,6,9,20,26,33,34]^ with frequent infiltration by neutrophils,^[14,30,32,35]^ lymphocytes,^[8,32,35,38]^ plasma cells,^[32,38]^ and giant cells.^[26,37,38]^ Occasionally, eosinophils^[14]^ and Langerhans cells^[38]^ were also observed.

Pituitary magnetic resonance imaging (MRI) plays a crucial role in lesion identification. Common MRI abnormalities include pituitary gland enlargement.^[4-8,15,17,21,25,26,28-30,33,35,36,38-45]^ Other findings included sellar masses,^[5,13,14,16,20,22,29,30,32,37,38,42,44-48]^ hypointense signal on T1-weighted sequences and hyperintense on T2-weighted sequences,^[7,15,17,39,41,45,46,49]^ infiltration or thickening of the infundibulum with heterogeneous contrast enhancement.^[5,12,32,43,45]^ About 12 of 91 patients had optic chiasm compression,^[5,8,21,22,27,32,33,37,38,46,48]^ often accompanied by vision field loss.^[21,32,33,37,46]^ In the MRI findings of our 2 cases, 1 case was associated with a congenital Rathke’s cleft cyst and pituitary enlargement, while the other case showed pituitary enlargement with heterogeneous contrast enhancement, which is consistent with the reported findings. In case 1, there was no significant change in the size of the pituitary cyst before and after treatment. It showed high signal intensity on both T1- and T2-weighted images, indicating the presence of a fluid with high protein concentration. The contrast-enhanced MRI showed no enhancement, suggesting it was a cyst rather than an adenoma or inflammatory nodule. Therefore, we consider the cystic structure in case 1 to be a congenital Rathke’s cleft cyst, unrelated to hypophysitis. Additionally, the minimal change in the size of the pituitary after treatment in case 1 may be due to the relatively large volume occupied by the Rathke’s cleft cyst. In case 2, the pituitary volume decreased after disease remission. Based on the literature regarding pathology, we believe that pituitary enlargement may be related to increased infiltration of inflammatory cells such as lymphocytes or local granuloma formation.

Pituitary dysfunction mostly affects the secreting of ADH, as DI presented in most cases.^[4-13,15,17,18,20-32,34-37,39-41,43-46,48-53]^ Anterior hormone insufficiency was also involved in more than half of PD with AAV, 49.33% of the patients present decrease of FLH-LH,^[4-6,8,12-14,20,22,25,27,30,32,39,40,42,43,49,51]^ 44.13% of the patients present decrease of thyroid-stimulating hormone,^[6-8,14,20,27,29,30,32,33,38-40,42-44,46,49,51,52]^ 52.38% of the patients present decrease of adrenocorticotropic hormone^[5,8,12,14,27,39,40,43,51]^ (despite those patients who are under Glucocorticoid treatment), 20.34% of the patients present decrease of GH,^[4,5,8,14,22,27,35,39,49]^ and 42.86% of the patients present decrease of PRL.^[4-8,10,15,21,25,29,30,33,37-39,41,42,45,49,52]^ DI presented in our 2 cases as well, and 1 case present decrease of PRL.

Most AAV with PD patients presented other organ lesions. Among them, ear, nose, throat,^[4-8,10,15,17,18,20-23,25-29,33,35,36,39,40,43-45,49,51,52]^ eye,^[5,7,8,10,12,15,18,21,23,28,30-33,37-39,44,46,50,51,53]^ pulmonary,^[4-8,15,17,18,20-22,25,28-30,34,36,40,41,44,46,49]^ and kidney^[4,6-10,18,21,25,29,36,39,44,46,49,51]^ were the most 4 frequently involved organs. Our first patient exhibited pituitary dysfunction (PD) as the initial presentation, accompanied by ear, nose, throat and musculoskeletal symptoms. In contrast, the second patient initially presented with nephritis and progressed to DI after 3 years.

For ANCA patterns, according to literatures, cANCA/PR3 (65.67%) was the main type of these patients,^[4,6,8-15,20-22,24,26-29,33,37,39-41,44,46,50,52,53]^ followed by pANCA/MPO (20.90%).^[4,7,8,23,29,35,36]^ Only 13.43% patients were serum negative AAV.^[4,8,30,32,38]^

The treatment approach for these patients primarily involved controlling ANCA-AAV activity alongside pituitary hormone replacement therapy. Immunosuppressive therapy was commonly used, with glucocorticoids, particularly prednisone, combined with cyclophosphamide, being the most frequently reported regimen. In 20 out of the 94 patients, higher doses of glucocorticoids were administered. When AAV relapsed or refractory cases occurred, other immunosuppressive agents such as rituximab (RTX), tacrolimus (FK506)/cyclosporine A (CsA), methotrexate, and MMF were often prescribed.

The majority of patients achieved systemic disease remission or improvement during follow-up; however, more than half continued to exhibit persistent pituitary dysfunction.

In conclusion, our case and accompanying literature review reveal the importance of considering ANCA-AAV in patients presenting with pituitary disfuction. When AAV patients present with symptoms such as oral dryness, polyuria, fatigue, oligomenorrhea, and somnolence, pituitary involvement should be considered and pituitary function-related tests and pituitary MRI should be performed to rule out the pituitary disfunction. Due to the possibility of optic chiasm compression in AAV patients with pituitary dysfunction, when patients present with tubular visual fields, visual abnormalities, or MRI findings suggesting optic chiasm involvement, it is important to complete vision field tests. Additionally, if needed, fundus fluorescein angiography should be performed to distinguish retinal vasculitis lesions and orbital MRI to rule out orbital granulomatous lesions. The latest EULAR^[54]^ and KDIGO^[55]^ guidelines recommend using glucocorticoids combined with rituximab or cyclophosphamide as the initial treatment for firsly diagnosed ANCA-AAV. In non-severe cases, MMF may serve as an alternative to cyclophosphamide, particularly in the MPO-ANCA subgroup. For maintenance therapy after achieving remission induction, rituximab or azathioprine combined with low-dose glucocorticoids are recommended. In our first case, due to initial treatment and possible concurrent pulmonary infection, a weaker immunosuppressant – mycophenolate mofetil – was chosen. Subsequently, the patient achieved disease remission. In our second case, because of disease relapse after initial treatment and the patient’s relatively young age, RTX was administered as immunosuppression after excluding infection, leading to remission again. In both cases, pituitary function was restored. Based on a review of the literature, we believe that in addition to hormone replacement therapy, effective immunosuppressive treatment for the underlying disease is also crucial in managing AAV-related pituitary dysfunction. Combination therapy with corticosteroids and stronger immunosuppressants such as RTX or cyclophosphamide has been used in most cases as an induction regimen for remission. However, good efficacy has also been observed with MMF and methotrexate in some patients. Therefore, we suggest that the selection of immunosuppressive agents for inducing remission should be based on the patient’s general condition, overall disease activity, and whether there is a concurrent infection, in order to balance treatment efficacy and potential side effects.

Supplemental digital content “Supplemental Figure” is available for this article (http://links.lww.com/MD/R146).

## Acknowledgments

The authors thank the patients and their family for their cooperation.

## Author contributions

**Conceptualization:** Xin Chen, Yan Du.

**Data curation:** Xin Chen, YiFan Xie, MengLi Xu, Chao Wang.

**Formal analysis:** Xin Chen.

**Funding acquisition:** Yan Du.

**Investigation:** YiFan Xie.

**Methodology:** YiFan Xie.

**Supervision:** Jing Xue.

**Validation:** YiFan Xie, MengLi Xu, Qi Cheng.

**Visualization:** MengLi Xu, Chao Wang.

**Writing – original draft:** Xin Chen.

**Writing – review & editing:** MengLi Xu, Qi Cheng, Jing Xue, Yan Du.

## Supplementary Material


