# Supplementary figures and images for: Pituitary involvement in ANCA-associated vasculitis: Case report and literature review
Source: Medicine (Baltimore). 2026 Jan 23;105(4):e47229. doi: 10.1097/MD.0000000000047229 (PMC12851694; doi:10.1097/MD.0000000000047229)

## Slide 1
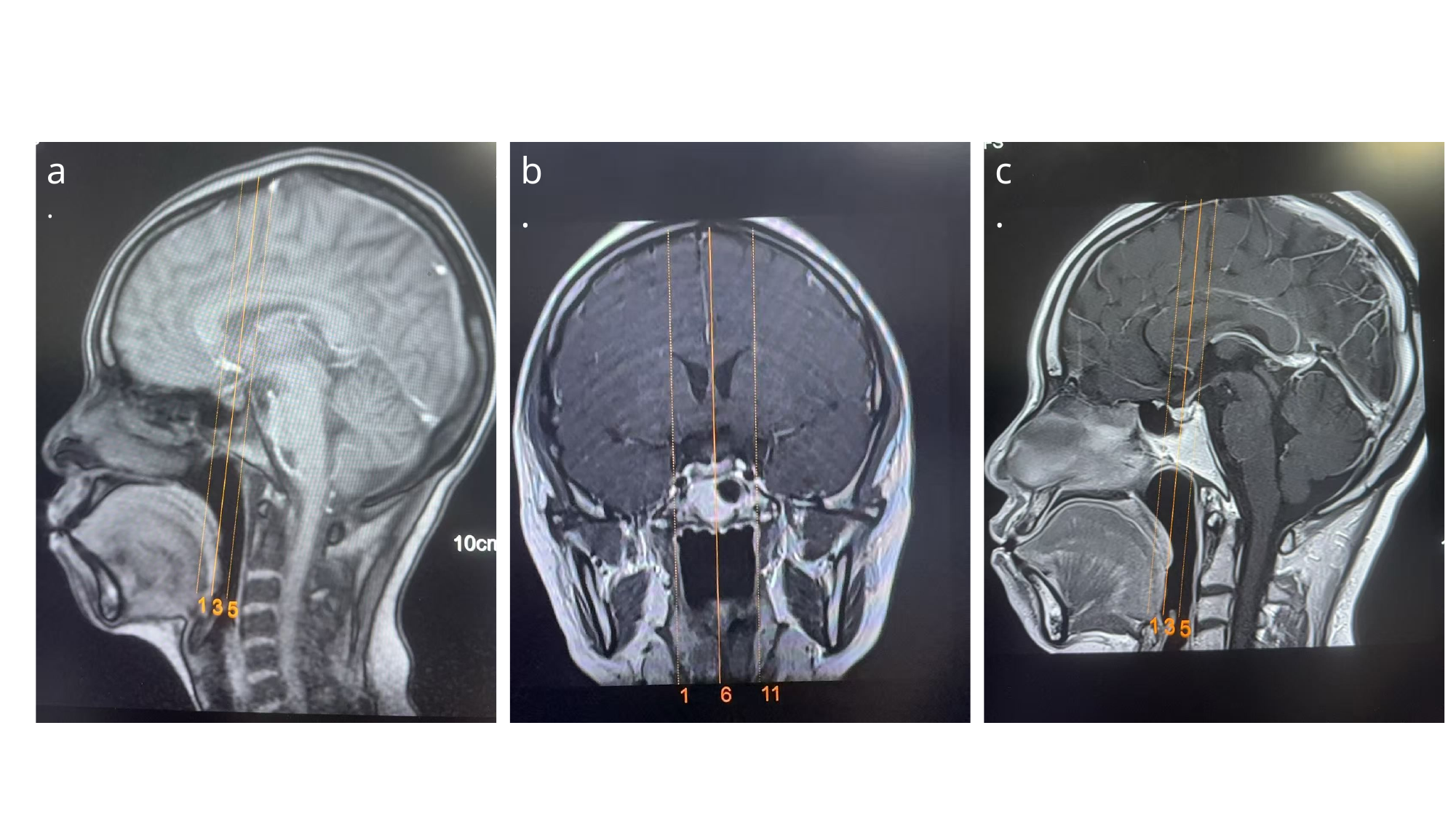

a.
b.
c.

Supplement: Supplementary file 1 [file medi-105-e47229-s001.pptx]
